# HMGB1 in severe soft tissue infections caused by *Streptococcus pyogenes*

**DOI:** 10.3389/fcimb.2014.00004

**Published:** 2014-01-30

**Authors:** Linda Johansson, Johanna Snäll, Parham Sendi, Anna Linnér, Pontus Thulin, Adam Linder, Carl-Johan Treutiger, Anna Norrby-Teglund

**Affiliations:** ^1^Department of Medicine, Center for Infectious Medicine, Karolinska InstitutetStockholm, Sweden; ^2^Division of Infection Medicine, Department of Clinical Sciences, Lund University HospitalLund, Sweden

**Keywords:** HMGB1, soft tissue infections, *Streptococcus pyogenes*, necrosis, inflammation

## Abstract

Extracellular High Mobility Group Box 1 (HMGB1) has been associated with acute and chronic inflammatory conditions. However, little is known about HMGB1 in necrotizing bacterial infections. We hypothesized that the local HMGB1 response is excessive in severe soft tissue infections (STIs), which are characterized by necrosis and hyperinflammation. To explore this, tissue biopsies were collected from patients with varying severity of *Streptococcus pyogenes* skin and STIs, including erysipelas, cellulitis, and necrotizing fasciitis. Tissue sections were immunostained for HMGB1, *S. pyogenes*, and inflammatory cell infiltrates and results quantified by acquired computerized image analysis (ACIA). HMGB1 expression increased in parallel to disease severity and was significantly higher in necrotizing fasciitis than in erysipelas (*p* = 0.0023). Confocal microscopy of sections co-stained for HMGB1 and cell markers revealed both extracellular and cytoplasmic HMGB1, the latter of which was found predominantly in macrophages. To further verify macrophages as main source of activation triggered HMGB1 release, human macrophages were infected with clinical *S. pyogenes* isolates. The results demonstrated infection triggered release of HMGB1. Dual staining's visualized HMGB1 in areas close to, but not overlapping, with neutrophils, indicating a potential chemotactic role. *In vitro* transmigration experiments showed a chemotactic effect of HMGB1 on neutrophils. The data furthermore provided *in vivo* support that HGMB1 may form immunostimulatory complexes with IL-1β. Taken together, the findings provide the first *in vivo* evidence that HMGB1 is abundant at the local site of severe bacterial STIs and its levels correlated to severity of infections; hence, indicating its potential value as a biomarker for tissue pathology.

## Introduction

High mobility group box protein 1 (HMGB1) is a ubiquitously expressed, highly conserved nuclear protein that is released either through active secretion by innate immune cells, enterocytes and hepatocytes or through passive release by injured, autophagic or necrotic cells (Bianchi et al., 1989; Tsung et al., 2007; Livesey et al., 2009; Skinner, 2010; Tang et al., 2010; Thomas and Stott, 2012). Extracellular HMGB1 has been reported to act as an alarmin with ability to activate the immune system leading to cell proliferation, adhesion, migration, and cytokine release (Harris et al., 2012). Several receptors have been shown to interact with HMGB1 including toll-like receptor 4 (TLR4), the receptor for advanced glycation end products (RAGE) and CD24-Siglec-10 (Lotze and Tracey, 2005; Tian et al., 2007; Chen et al., 2009). Studies have suggested differential responses depending on receptor engagement, with TLR4 being involved in cytokine release (Yang et al., 2010) whereas RAGE is involved in cell migration (Penzo et al., 2010). Importantly post-translational modifications, including acetylation, phosphorylation, methylation and redox state of the cysteine residues, influence receptor interactions, and thereby, elicited responses (Harris et al., 2012; Venereau et al., 2012; Yang et al., 2012).

HMGB1 has been implicated in a variety of clinical conditions including arthritis, sepsis and chronic kidney disease (Harris et al., 2012). In sepsis, murine experimental data demonstrated a critical role of HMGB1 as a late mediator contributing to mortality (Wang et al., 1999). Subsequently, elevated levels of HMGB1 have been readily detectable in circulation of patients with severe sepsis and septic shock (Sunden-Cullberg et al., 2005). However, little is known about local HMGB1 responses in acute necrotic bacterial soft tissue infections (STIs), such as necrotizing fasciitis. These are life-threatening infections where the vast majority of patients require intensive care and extensive surgical interventions. Despite modern medicine, the mortality associated with necrotizing soft tissue infections (NSTIs) is high, often exceeding 30% (Anaya et al., 2005; Marwick et al., 2011). Although a number of bacteria can cause NSTI, the main causative microbes are *Streptococcus pyogenes* and *Staphylococcus aureus* (McHenry et al., 1995). Here we set out to gain insight into events occurring in the microenvironment at the infected tissue site by delineating HMGB1 responses in acute necrotic STIs caused by *Streptococcus pyogenes*. Analyses of tissue biopsies from patients with varying severity of *S. pyogenes* infections revealed a significant correlation between increased HMGB1 and severity of tissue infection.

## Materials and methods

### Patient material

The biopsy material from severe streptococcal infections has been described previously in (Thulin et al., 2006). The material included snap-frozen tissue biopsies (*n* = 25) collected from six patients with necrotizing fasciitis (*n* = 22) or cellulitis (*n* = 3), caused by *S. pyogenes* of varying serotypes (Table 1). Biopsies received a clinical grade at the time of sampling (grade 1: distal tissue not visually inflamed; *n* = 5, grade 2: inflamed tissue including cellulitis, fasciitis, and necrotizing fasciitis; *n* = 20) (Thulin et al., 2006). Snap-frozen punch biopsies collected from patients with erysipelas (*n* = 11) were also included (Linder et al., 2010).

**Table 1:** **Characteristics of tissue biopsy material**.

**Patient ID**	**Diagnosis**	**GAS serotype**	**Day of sampling ^a^**	**Type of tissue**
8612	STSS/NF	M28	2	Subcutaneous
			2	Muscle
			4	Fascia
			4	Muscle
			4	Fascia
8611	STSS/NF	M4	1	Fascia
			1	Subcutaneous
			2	Fascia
			2	Subcutaneous
			2	Muscle
			2	Fascia
			2	Subcutaneous
			3	Fascia
			3	Muscle
8489	STSS/NF	M1	1	Fascia
			1	Muscle
			4	Subcutaneous
8157	STSS/NF	M1	3	Muscle
			9	Muscle/fascia
5626	NF	M3	1	Muscle
			3	Muscle
			6	Fascia
8271	Cellulitis	M1	1	Subcutaneous
			3	Muscle
			3	Muscle
1	Erysipelas	n.a.	3	Skin
3	Erysipelas	n.a.	2	Skin
4	Erysipelas	n.a.	2	Skin
5	Erysipelas	n.a.	1	Skin
6	Erysipelas	n.a.	2	Skin
7	Erysipelas	n.a.	3	Skin
8	Erysipelas	n.a.	1	Skin
9	Erysipelas	n.a.	3	Skin
10	Erysipelas	n.a.	1	Skin
11	Erysipelas	n.a.	2	Skin
12	Erysipelas	n.a.	2	Skin

GAS, Group A Streptococcus; STSS, streptococcal toxic shock syndrome; NF, necrotizing fasciitis; n.a., not available.

a *After onset of diagnosis*.

Plasma samples collected at admission from patients with Streptococcal Toxic Shock Syndrome (STSS) were included (*n* = 14) (Darenberg et al., 2003) as well as plasma samples from 14 healthy volunteers (controls).

All samples were obtained from the patients after written informed consent and from healthy volunteers, according to protocols approved by the ethics committee at respective University and in accordance with the Declaration of Helsinki Principles.

### Immunostainings of tissue biopsy specimen

Biopsies were cryosectioned (8 μm), fixed in 2% formaldehyde and immunostained as previously described (Norrby-Teglund et al., 2001; Thulin et al., 2006; Johansson et al., 2009). The immunohistochemical staining was modified to include an initial blocking step with 10% FCS in Earl's balanced salt solution and 0.1% saponin at room temperature for 30 min. The brown color reaction was developed by diaminobenzidine (DAB-kit from Vector Lab Inc.). The staining pattern of HMGB1 in the biopsies revealed both intracellular and extracellular HMGB1; however, not all cells stained positive despite the fact that this is a nuclear factor. This has also been noted by others (Ulfgren et al., 2004), and is likely due to technical limitations in identification of nuclear HMGB1, potentially through insufficient antibody access to the nuclei. The immunohistochemically stained sections were analyzed by acquired computerized image analysis (ACIA), which has proven to be a robust method for semiquantitative assessment of cell infiltrates and inflammatory markers in single cells and tissue (Norrby-Teglund et al., 2001; Thulin et al., 2006; Johansson et al., 2008). Analyzed cell area (defined by the haematoxylin counterstaining) ranged from 0.2 × 10^5^ to 7.9 × 10^5^ μm^2^ for deep tissue biopsies and 9.6 × 10^5^ to 7 × 10^6^ μm^2^ for erysipelas biopsies. The results are presented as ACIA value, i.e., percent positively stained area × mean intensity of positive staining.

Single and dual immunofluorescence stainings were performed as previously described (Thulin et al., 2006) and visualized using either a Leica confocal scanner TCS2 AOBS with an inverted Leica DMIRE2 microscope (Wetzlar, Germany) or a Nikon A1R confocal microscope (Nikon Instruments, Amstelveen, the Netherlands).

The following antibodies were used: anti-CD68, anti-neutrophil elastase, anti-mast cell tryptase (DAKO, Glostrup, Denmark), biotinylated anti-IL-1β/IL-1F2, anti-CXCL12 (R&D systems, Minneapolis, MN), and anti-CD1a (BD Biosciences Pharmingen, San Diego, CA). HMGB1 was initially identified using a polyclonal anti-HMGB1 from BD Biosciences Pharmingen (556528, BD Diagnostics, San Diego, CA) or anti-human HMGB1 (ab18256, Abcam, Cambridge, UK). *Streptococcus pyogenes* were identified with a polyclonal rabbit antiserum specific for the Lancefield group A carbohydrate (Difco, BD Diagnostics, Sparks MD). Biotinylated secondary antibodies included goat-anti-mouse IgG and goat-anti-mouse IgG (both from Vector Laboratories, Burlingame, CA).

### Bacterial strains

Two clinical M3 isolates of *S. pyogenes*, obtained during the collection of the Canadian patient material described above, were grown over night (16–18 h) in Todd-Hewitt broth complemented with 1.5% yeast extract. A clinical isolate of *Escherichia coli*, obtained from a sepsis patient, was also included in the study and was grown over night in Luria Broth medium. Before infection the bacterial cultures were centrifuged at 2500 rpm for 10 min and resuspended in RPMI 1640 cell culture medium supplemented with 5% heat inactivated FCS, 10 mM L-glutamine, and 25 mmol/L HEPES (all from Thermo Scientific, Hyclone).

### Infection of primary human monocyte-derived macrophages

Human monocyte-derived macrophages were isolated form healthy blood donors using RosetteSep (Stemcell Technology) and Lymphoprep (Axis-Shield) gradient centrifugation, as previously described (Hertzen et al., 2010). After isolation, the isolated cells were resuspended in RPMI 1640 cell culture medium supplemented with 5% heat inactivated FCS, 10 mM L-glutamine, Penicillin (100 U/ml)/Streptomycin (100 ug/ml) solution, and 25 mmol/L HEPES (all from Thermo Scientific, Hyclone) as well as 50 ng/ml of Macrophage colony stimulating factor (Immunotools). Cells were seeded at a concentration of 1 × 10^6^ cells/ml in 6-well low adherence tissue culture plates (Corning) and cultured for 7–8 days to obtain macrophages. At day 8 cells were washed with phosphate buffered saline (PBS; Sigma-Aldrich, St Louis, MO), harvested and resuspended in RPMI 1640 cell culture medium supplemented with 5% heat inactivated FCS, 10 mM L-glutamine and 25 mmol/L HEPES. Cells were seeded in 24-well tissue culture plate, containing a sterile coverslip at the bottom of each well, at a concentration of 1 × 10^6^ cells/ml and incubated over night at 37°C with 5% CO_2_. Cells were then infected with *S. pyogenes* or *E. coli*, at a multiplicity of infection (MOI) of 1–11 bacteria per cell. Four hours post infection cell culture supernatants were collected and centrifuged at 2500 rpm for 10 min, aliquoted and stored at −20°C. The cells, situated on coverslips were fixed with 2% paraformaldehyde for 15 min at room temperature. After fixation, cells were washed with PBS, dried and stored at −20°C.

### HMGB-1 ELISA

HMGB-1 was measured by ELISA (Shino-Test Corporation, Tokyo, Japan) according to manufacturer's instructions. The range of detection was 2.5–80 ng/ml. All samples were analyzed in duplicates.

### IL-8 Luminex

Plasma level of IL-8 was determined by Luminex IL8 human singleplex bead kit (Invitrogen, Paisley, UK), and the Luminex (100) instrument (Luminex, Invitrogen, Paisley, UK). All samples were analyzed in duplicate.

### Western blot

The positive control recombinant HMGB1 (provided in dithiothreitol (DTT); R&D Systems, Minneapolis, MN) and tissue biopsies (*n* = 2) (12 sections, 8 μm thick) suspended in PBS were incubated with NuPAGE SDS loading buffer (Life Technologies, Carlsbad, CA) at 90°C for 15 or 25 min, respectively. Proteins were separated using a 12% NuPAGE Novex Bis-Tris gel (Life Technologies, Carlsbad, CA) and transferred onto nitrocellulose membrane using the iBlot system (Life Technologies, Carlsbad, CA). The membrane was blocked using 5% dry milk powder and 0.1% Tween-20 PBS saline. HMGB1 was detected using an anti-HMGB1 antibody (Sigma Aldrich, St. Louis, MO) in combination with a horseradish-peroxidase labeled anti-mouse antibody (GE Healthcare, Little Chalfont, UK), and an electrochemiluminescence kit (Thermo Scientific, Waltham, MA). Both the oxidized or reduced forms of HMGB1 were detected under these conditions in sizes corresponding to that reported previously (Venereau et al., 2012). It should be noted that the recombinant protein has a molecular weight of 24.9 kDa but separates as a 30–36 kDa protein in SDS-PAGE according to the manufacturer's specification.

### Chemotaxis assay

Chemotactic effect of HMGB1 was measured using a transwell-based assay as detailed in Berthelot et al. (2012). Whole-blood was chosen as a previous report showed that neutrophil isolation methods might affect cell motility (Sroka et al., 2009). In short, 600 μl of PBS with 1% human serum and recombinant HMGB1 or IL8 (R&D Systems, Minneapolis, MN; both supplied in preparations with endotoxin levels <1 EU/μg) was added to the basolateral side of a 3 μm pore-size polycarbonate transwell membrane (Coring incorporated, Corning, NY). One hundred microliters of human whole blood diluted 1/10 in PBS was added to the membranes apical side, and cells left to migrate for 2 h in 37°C. Migrated cells were collected and stained with anti-human CD14 PE and anti-human CD15 FITC (BD Bioscience, San Jose, CA). The number of migrated neutrophils was determined using a BD LSRII Fortessa cell analyser (BD Bioscience, San Jose, CA) in combination with CountBright Absolute Counting Beads (Molecular Probes Inc., Eugene, OR), and analyzed using the FlowJo software version 9.5.3 (Tree Star, Ashland, OR).

## Results

### Level of HMGB1 response correlates to severity of *S.pyogenes* tissue infections

**Figure 1:**
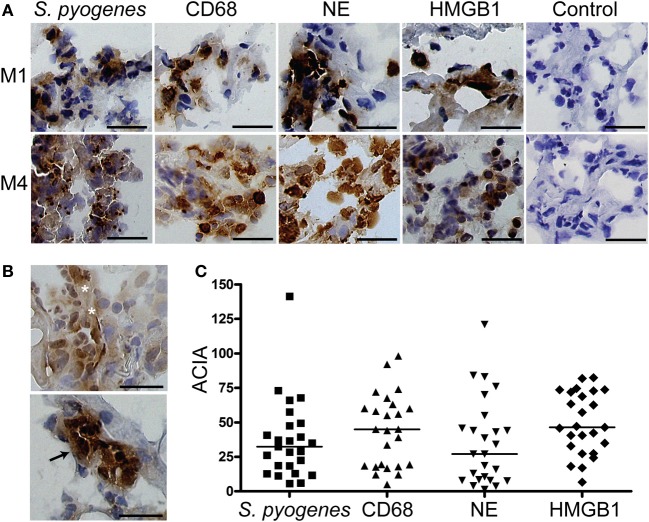
**HMGB1 at the local site of infection**. Snap-frozen tissue biopsies (*n* = 25) from patients with severe soft tissue infections caused by *S. pyogenes* were immunohistochemically stained for *S. pyogenes*, macrophages identified by CD68-positivity, neutrophils identified by neutrophil elastase positivity, and HMGB1. A control staining where the primary antibody was omitted was also performed (control). **(A)** Representative immunohistochemically stained biopsies taken from the site of infection at day 1 from two patients (patient 8271, top panel, and patient 8611, bottom panel) (Table 1). Positive stainings for cell markers and for HMGB1 are indicated by a brown color, and all cellular nuclei are stained blue by haematoxylin. Bars indicate 50 μm. **(B)** Staining's for HMGB1 revealed both a diffuse (top picture, indicated by white stars) and a distinct intracellular staining (bottom picture, indicated by the black arrow). Bars indicate 50 μm. **(C)** Image analysis values for *S. pyogenes*, macrophages (CD68), neutrophils (NE), and HMGB1 in the different biopsies were obtained by ACIA; for details see Materials and Methods. The horizontal lines denote median.

**Figure 2:**
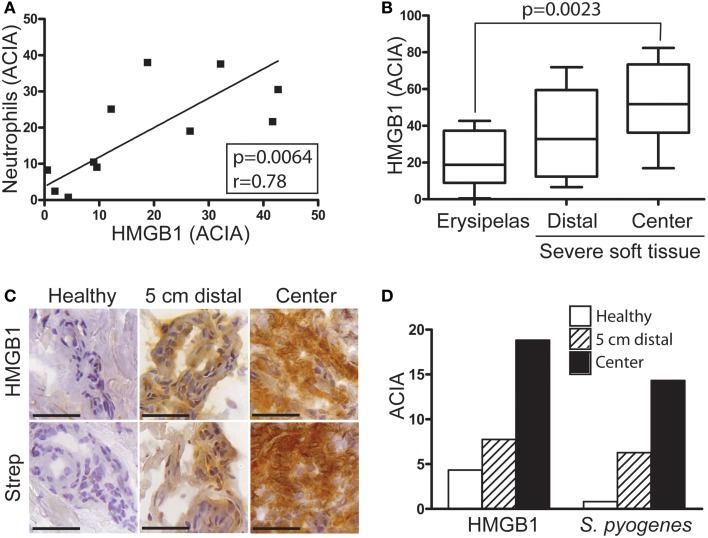
**HMGB1 correlates with severity of infection**. Snap-frozen tissue biopsies (*n* = 11) from patients with erysipelas were immunohistochemically stained for *S. pyogenes*, neutrophils identified by neutrophil elastase positivity and HMGB1. **(A)** Correlation between neutrophil infiltration in relation to HMGB1 ACIA value (for details see Materials and Methods) in erysipelas biopsies. Significant correlation, as determined by Pearson's correlation test, is indicated by *p*- and *r*-values. **(B)** Image analysis data of HMGB1 in tissue from patients with erysipelas were compared with biopsies from patients with severe streptococcal soft tissue infection of clinical grade 1 and 2 (for details see Materials and Methods). Statistically significant differences were determined by 1way ANOVA, Kruskal-Wallis; *p* = 0.0023. **(C)** Representative immunohistochemical pictures of erysipelas tissue biopsies collected at the inflamed center, 5 cm outside the lesion and from the healthy leg. Bars indicate 50 μm. **(D)** Acquired computerized image analysis values of the whole tissue biopsy.

First we sought to determine the presence of HMGB1 at the local tissue site in *S. pyogenes* infected patients. To this end, a tissue material collected from patients with varying severity of streptococcal tissue infections, i.e., mild erysipelas (*n* = 11), to more severe STIs including severe cellulitis and necrotizing fasciitis (*n* = 6) (Table 1), were immuno-histochemically stained for HMGB1, bacterial load, and inflammatory cells. Stainings were evaluated by microscopy and *in situ* image analysis. HMGB1 was detected in all analyzed biopsies (11 erysipelas and 25 severe STIs) and demonstrated both a diffuse, supposedly secreted HMGB1, as well as distinct intracellular staining (evident in some but not all cells) (Figures 1A,B). In agreement with our previous publication (Thulin et al., 2006), in severe STIs all biopsies displayed high bacterial burden and heavy infiltration of both macrophages and neutrophils (Figures 1A,C). Although HMGB1 was commonly found in the areas with dense bacteria load and inflammatory cell infiltrates (Figure 1A), neither bacterial load, nor phagocytic cellular infiltration correlated significantly with amount of HMGB1 (data not shown). It seems likely that this lack of association might be due to the lack of tissue integrity caused by the high degree of necrosis present in severe STI biopsies. In fact in erysipelas where no necrosis is present, HMGB1 significantly correlated with the neutrophil infiltration (*r* = 0.78; *p* = 0.0064) (Figure 2A), but not with macrophage infiltration or bacterial load (data not shown). The latter is not surprising as the bacterial load is substantially lower in erysipelas than in severe STIs (Linder et al., 2010).

Importantly, semiquantitative assessment of HMGB1 at the infected site in patients with erysipelas compared to severe STIs revealed an increase in parallel to disease severity (*p* = 0.0023) (Figure 2B). The lowest values were noted in erysipelas biopsies, followed by biopsies from severe STIs taken in distal visually not inflamed areas, and the highest were found at the center of infection in biopsies from STI (Figure 2B). Similarly, analyses of biopsies taken from a patient with erysipelas at the center of infection, distal (5 cm outside the lesion) and healthy region (other non-infected leg) demonstrated that both HMGB1 and bacterial load increased with more involved inflamed tissue (Figures 2C,D).

**Figure 3:**
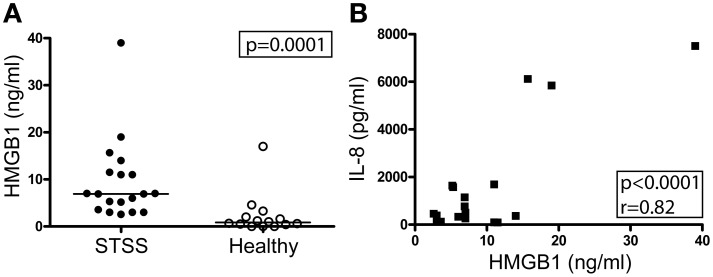
**Elevated HMGB1 in circulation in patients with STSS**. HMGB1 and IL-8 levels in plasma collected on the day of inclusion were measured with ELISA and Luminex respectively, for details see Materials and Methods. Shown in **(A)** is systemic HMGB1 in STSS patients (*n* = 19) and healthy controls (*n* = 14). Statistically significant differences were determined by two-tailed *t*-test; *p* = 0.0001. Shown in **(B)** is systemic HMGB1 and IL-8 in patients with STSS (*n* = 19 patients). Significant correlation, as determined by Pearson's correlation test, indicated by *p*- and *r*- values.

This marked local HMGB1 response led us to explore systemic HMGB1 levels in a cohort of patients with the most severe manifestation of invasive *S. pyogenes* infections, namely STSS. Plasma levels of HMGB1 were measured at the day of inclusion. Significantly elevated levels of HMGB1 were detected in patients as compared to healthy controls (Figure 3A, *p* = 0.0001). For comparison, all STSS samples were also analyzed for IL-8, a commonly used marker for systemic inflammation. A significant correlation was found between the two markers (*r* = 0.82; *p* < 0.0001) (Figure 3B).

### HMGB1 in relation to inflammatory cells and innate immune factors *in vivo* and *in vitro*

**Figure 4:**
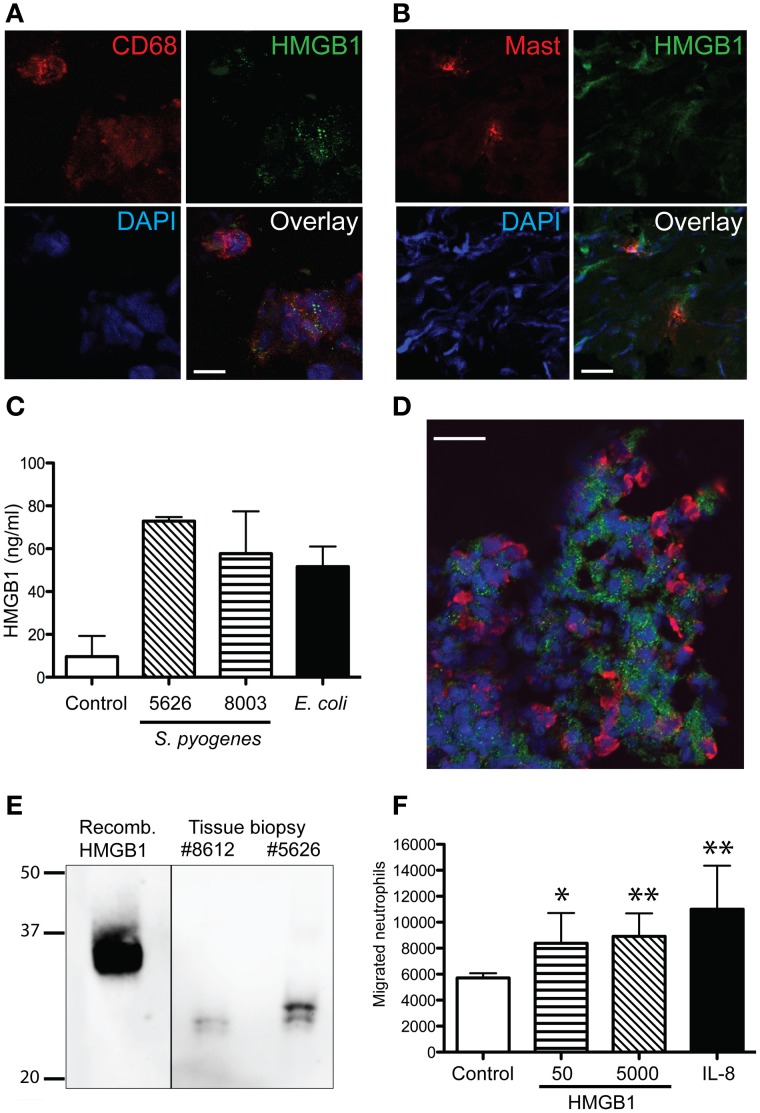
**Cellular source of HMGB1 at the site of infection and chemotactic effect of HMGB1 on neutrophils**. The images depicts a representative cryosections of tissue biopsies from patients with erysipelas, immunofluorescently stained for **(A,B,D)** HMGB1 (green) in combination with **(A)** macrophages (red) identified by CD68-positivity, **(B)** mast cells tryptase, or **(D)** neutrophils (red) identified by neutrophil elastase. Note the granular staining of HMGB1 **(A,D)**. Cell nuclei are stained blue with DAPI. Bars indicate 10 μm **(A)** and 20 μm **(B,D)**. **(C)** Human monocyte-derived macrophages were isolated from healthy blood donors and infected with *S. pyogenes* or *E. coli* at an MOI of 1–11 for 4 h. Supernatants were collected and analyzed for HMGB1 using an ELISA. Uninfected macrophages were used as control. The graph shows the results obtained from two different donors. **(E)** Western blot of recombinant HMGB1 and protein extracts from patient tissue biopsies (patient 5626 and 8612). Proteins were separated using a 12% NuPAGE Bis-Tris gel and visualized using a monoclonal anti-HMGB1 antibody. **(F)** The chemotactic effect of HMGB-1 on primary neutrophils was measured using a transwell assay system where neutrophils were left to migrate against 50 and 5000 ng/ml HMGB-1, respectively, or 25 ng/ml IL-8 for 2 h. Spontaneous migration was measured against 1% human serum in PBS (control) (for details, see Materials and Methods). Values represent means ± s.e.m. (*n* = 5) and statistical difference between the control and each respective sample as determined using the Mann-Whitney test, ^*^p < 0.05, ^**^p < 0.01.

To further characterize HMGB1 at the local site of infection in relation to inflammatory cells dual immunofluorescence stainings were performed, using anti-HMGB1 combined with antibodies against various cell markers, including CD68 (macrophages), neutrophil elastase, mast cell tryptase, and CD1a (Langerhans cells). In both erysipelas and severe STI, confocal microscopy analyses revealed that HMGB1 in tissue cells display a characteristic vesicular staining pattern (Figures 4A,D); a pattern consistent with active nuclear translocation of HMGB1 to the cytoplasm (Gardella et al., 2002). Importantly, the only two cell types in the tissue that demonstrated this intracellular HMGB1 were macrophages (Figure 4A) and mast cells (Figure 4B). The latter of which constituted only a minor part of the HMGB1 positive population, due to the low frequency of mast cells in skin and soft tissue. Thus, the data demonstrates that macrophages are the major source of active released HMGB1 at the infected tissue site. To further support this finding, human monocyte-derived macrophages were infected with two different clinical strains of *S. pyogenes* for 4 h. The levels of HMGB1 in the cell supernatant were measured with an ELISA. HMGB1 was readily detected in the culture medium of infected macrophages compared with non-infected macrophages (Figure 4C).

Co-staining of neutrophils and HMGB1 revealed that the neutrophils were present in areas of HMGB1, but not co-localizing with HMGB1. In fact, in some areas neutrophil infiltrates were found to surround HMGB1 positive cell populations (Figure 4D). As previous studies have attributed the chemotactic activity to the redox state of HMGB1 (Harris et al., 2012), we next sought to determine the form of HMGB1 in the tissue. Therefore, protein extracts of tissue biopsy sections were separated on a SDS-PAGE gel under non-reducing conditions and analyzed by Western blot. Detection of HMGB1 in tissue sections revealed two bands with a similar size and shift that was previously reported corresponding to the reduced chemotactic all-thiol-HMGB1 and the pro-inflammatory disulfide-HMGB1 (Figure 4E) (Venereau et al., 2012). The effect of HMGB1 on neutrophil motility was tested in a human whole-blood transwell migration assay using different concentrations of HMGB1. Both low (50 ng/ml) and high (5000 ng/ml) concentrations induced a significant neutrophil migration (*p* < 0.05); almost to the same extent as the positive control IL-8 (Figure 4F).

**Figure 5:**
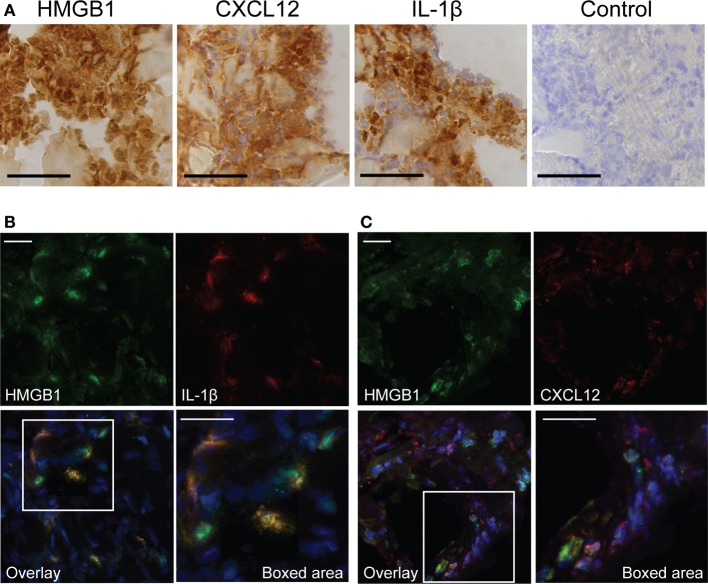
**Presence of pro-inflammatory mediators at the local site of tissue infection**. The figure shows representative images of a snap-frozen cryo-sectioned biopsy, from a patient with severe streptococcal soft tissue infection. **(A)** Tissue sections were immunohistochemically stained for HMGB1, CXCL12, and IL-1β. A control staining, omitting the primary antibody was also made (control). Bars indicate 50 μm. Snap frozen tissue biopsies were also immunofluorescently stained for HMGB1 (green) in combination with **(B)** IL-1β (red) or **(C)** CXCL12 (red). Cell nuclei were stained blue with DAPI. Bars indicate 20 μm.

HMGB1 is able to form immunostimulatory complexes *in vitro* among others with IL-1β (Wahamaa et al., 2011) and CXCL12 (Schiraldi et al., 2012). To address the potential of such complexes to form *in vivo*, severe streptococcal tissue biopsies were immunohistochemically stained for CXCL12, IL-1β, and HMGB1. Our data showed both CXCL12 and IL-1β in the tissue, and these stainings coincided with areas that had high HMGB1 expression (Figure 5A). Furthermore, sections were immunofluorescently stained for HMGB1 in combination with either CXCL12 or IL-1β. Confocal microscopy analyses revealed HMGB1 overlapping with IL-1β (yellow areas, Figure 5B) suggesting co-localization. In contrast, this staining pattern was not noted with HMGB1 and CXCL12. The two proteins appeared in the same area but did not overlap (Figure 5C).

## Discussion

Despite HMGB1's direct link to necrosis and pro-inflammatory actions (Harris et al., 2012), there are, to the best of our knowledge, no clinical data to support these mechanistic actions in necrotizing bacterial infections. Here, we utilized biopsies from patients with skin and STIs caused by *S. pyogenes* to assess the presence of HMGB1 at the local infected site of infections characterized by inflammation and necrosis. Microscopy analyses revealed HMGB1 in all investigated biopsies, and notably, the levels correlated with severity of infection. For example, higher amounts of HMGB1 were found in biopsies from severe STIs as compared to erysipelas, as well as in the center as compared to distal site of infection. Per definition, necrotizing fasciitis is characterized by tissue necrosis. Intuitively, the HMGB1 noted at the tissue site is likely, at least in part, due to passive release from necrotic cells. However, our vesicular staining pattern of HMGB1 in the infiltrating immune cells indicates that there is also an active release of HMGB1 predominantly from macrophages, which likely involves the non-classical vesicle-mediated secretory pathway (Gardella et al., 2002). This was further strengthened by *in vitro* infection of human monocyte-derived macrophages with *S. pyogenes* resulted in a release of HMGB1.

In both erysipelas and soft tissue biopsies, infiltrates of neutrophils, and macrophages were present. In agreement with our previous publications (Thulin et al., 2006; Johansson et al., 2009; Linder et al., 2010), the severe STI biopsies showed massive cellular infiltrates, substantially higher than in erysipelas. Co-stainings of cellular markers and HMGB1 revealed a pattern where neutrophils were found close to, and in part, surrounding areas with HMGB1. As HMGB1 has been attributed chemotactic properties (Venereau et al., 2013), it is tempting to assume that this reflects a chemotactic response *in vivo* in infected tissue. A recent report suggested that the chemotactic effect is concentration dependent, and that the migration rate of neutrophils may be impaired or augmented by low or high HMGB1 concentrations, respectively (Berthelot et al., 2012). In contrast, in this study chemotactic responses were noted both at the low (50 ng/ml) and high (5000 ng/ml) concentrations. The experimental set-up, including HMGB1 concentrations, was similar to the previous report, but the source of HMGB1 differed. Due to limited tissue material, HMGB1 concentrations could not be directly quantified at the tissue site. However, our analyses of plasma from patients with STSS demonstrated HMGB1 levels ranging from 3–39 ng/ml. It seems likely that the levels at the local tissue site may exceed those found in circulation, especially considering the high image analyses values obtained in the tissue biopsies.

Recently, attention has been given to the function of HMGB1 as an alarmin that forms immunostimulatory complexes with cytokines and chemotactic factors (Harris et al., 2012; Schiraldi et al., 2012). These complexes, such as those formed with IL-1β and CXCL12, have *in vitro* been shown to be superior in their inflammatory or chemotactic properties. In our analysis, staining for IL-1β and CXCL12 showed that both factors are present in the tissue. Moreover, in the case of IL-1β a substantial overlay with HMGB1 was noted, providing proof-of-principle support for formation of these complexes *in vivo* given the close proximity of the factors in the infected tissue. Such complexes could theoretically contribute to the tissue destruction by augmentation of inflammation and leukocyte migration and thus, to disease pathogenesis.

## Author contributions

Experimental design (Linda Johansson, Anna Norrby-Teglund, Carl-Johan Treutiger, Pontus Thulin, Johanna Snäll, Anna Linnér, Parham Sendi, Adam Linder), acquisition of experiments (Linda Johansson, Johanna Snäll, Anna Linnér, Parham Sendi, Pontus Thulin, Adam Linder), data analysis (Linda Johansson, Johanna Snäll, Anna Linnér, Parham Sendi, Pontus Thulin, Anna Norrby-Teglund), and interpretation of data (Linda Johansson, Johanna Snäll, Anna Linnér, Parham Sendi, Pontus Thulin, Anna Norrby-Teglund, Carl-Johan Treutiger, Adam Linder). Manuscript writing (Linda Johansson, Anna Norrby-Teglund, Johanna Snäll, Carl-Johan Treutiger, Parham Sendi) and critical revision (Pontus Thulin, Carl-Johan Treutiger, Adam Linder). Final approval of the version to be published as well as agreement to be accountable for all aspects of the work in ensuring that questions related to the accuracy or integrity of any part of the work are appropriately investigated and resolved (Linda Johansson, Anna Norrby-Teglund, Johanna Snäll, Anna Linnér, Pontus Thulin, Parham Sendi, Adam Linder, Carl-Johan Treutiger).

### Conflict of interest statement

The authors declare that the research was conducted in the absence of any commercial or financial relationships that could be construed as a potential conflict of interest.
